# Inhibitors of the Interferon Response Enhance Virus Replication *In Vitro*

**DOI:** 10.1371/journal.pone.0112014

**Published:** 2014-11-12

**Authors:** Claire E. Stewart, Richard E. Randall, Catherine S. Adamson

**Affiliations:** School of Biology, University of St Andrews, Fife, Scotland, United Kingdom; University of Tennessee Health Science Center, United States of America

## Abstract

Virus replication efficiency is influenced by two conflicting factors, kinetics of the cellular interferon (IFN) response and induction of an antiviral state versus speed of virus replication and virus-induced inhibition of the IFN response. Disablement of a virus's capacity to circumvent the IFN response enables both basic research and various practical applications. However, such IFN-sensitive viruses can be difficult to grow to high-titer in cells that produce and respond to IFN. The current default option for growing IFN-sensitive viruses is restricted to a limited selection of cell-lines (e.g. Vero cells) that have lost their ability to produce IFN. This study demonstrates that supplementing tissue-culture medium with an IFN inhibitor provides a simple, effective and flexible approach to increase the growth of IFN-sensitive viruses in a cell-line of choice. We report that IFN inhibitors targeting components of the IFN response (TBK1, IKK2, JAK1) significantly increased virus replication. More specifically, the JAK1/2 inhibitor Ruxolitinib enhances the growth of viruses that are sensitive to IFN due to (i) loss of function of the viral IFN antagonist (due to mutation or species-specific constraints) or (ii) mutations/host cell constraints that slow virus spread such that it can be controlled by the IFN response. This was demonstrated for a variety of viruses, including, viruses with disabled IFN antagonists that represent live-attenuated vaccine candidates (Respiratory Syncytial Virus (RSV), Influenza Virus), traditionally attenuated vaccine strains (Measles, Mumps) and a slow-growing wild-type virus (RSV). In conclusion, supplementing tissue culture-medium with an IFN inhibitor to increase the growth of IFN-sensitive viruses in a cell-line of choice represents an approach, which is broadly applicable to research investigating the importance of the IFN response in controlling virus infections and has utility in a number of practical applications including vaccine and oncolytic virus production, virus diagnostics and techniques to isolate newly emerging viruses.

## Introduction

Virus infection triggers the cellular interferon (IFN) response to produce Type 1 IFN's alpha and beta (IFNα/β). Secreted IFNα/β can stimulate the JAK-STAT pathway in an autocrine or paracrine manner to activate hundreds of IFN-stimulated genes (ISGs), many of which have antiviral activities that elicit an antiviral state [1]. Although the IFN system constitutes a powerful antiviral response, it rarely works to full capacity because virus-encoded IFN antagonists circumvent it [1]. Manipulation of a virus's capacity to circumvent the IFN response enables both basic research and various practical applications. For example, genetic engineering has facilitated rational design of live-attenuated vaccines, where a common approach is to disable a virus's IFN antagonist thereby restricting its ability to circumvent the IFN response [2]–[8]. The rationale being that IFN antagonists are typically dispensable for virus replication in cell culture but are required for virulence *in vivo* and thus the vaccine will mimic natural infection in stimulating the immune system but without causing disease. Knockout of viral IFN antagonists is also a method of engineering viruses to specifically target cancer cells for oncolytic virotherapy [9], [10]. The rationale exploits the fact that tumorigenesis can result in impairment of innate immune responses, therefore viruses that no longer counteract the IFN response are often able to propagate in tumor cells but not normal cells and thus mediate tumor-specific killing.

Despite the advantages of disabling a virus's IFN antagonist, it can be difficult to grow such IFN-sensitive viruses to high-titer in tissue culture cells that produce and respond to IFN [11]. The current default option for growing such IFN-sensitive viruses is largely restricted to a very limited selection of cell-lines (e.g. Vero cells) that have lost their ability to produce IFN [12], [13]. However, many viruses do not grow efficiently in these cells, presumably due to other host cell constraints on virus replication [11]. To tackle this limitation, we have previously engineered cell-lines to no longer produce or respond to IFN by constitutive expression of Npro from Bovine Viral Diarrhea Virus (BVDV-Npro) which blocks IFN induction by targeting IRF3 for proteasome-mediated degradation [14] or constitutive expression of the parainfluenza type 5 virus V protein (PIV5-V), which blocks IFN signaling by targeting STAT1 for proteasome-mediated degradation [11]. In these engineered IFN incompetent cells vaccine candidate viruses and slow-growing wild-type viruses formed bigger plaques and grew to increased titers [11], demonstrating the potential use of these cell-lines for the applications described above. In addition such IFN incompetent cell-lines can be useful in virus diagnostics, isolation of newly emerging viruses and basic research [11]. However, genetically engineering cell-lines is time consuming and their use creates regulatory problems for vaccine manufacturers. We hypothesize that small molecule inhibitors of the IFN response would offer a simple and flexible solution, as an effective inhibitor could easily supplement the tissue culture medium of cell-lines of choice.

## Materials and Methods

### Inhibitors, viruses and cells

Inhibitors of the IFN response (BX795, MRT68844, MRT67307, TPCA-1, Cyt387, AZD1480, Ruxolitinib, Tofacitinib) were prepared as 10 mM stocks in dimethyl sulfoxide (DMSO) and used at the indicated concentrations. Inhibitors were purchased from Selleck Chemicals except MRT68844 and MRT67307, which were gifts of MRC-Technology and Philip Cohen (University of Dundee) respectively. Cell-lines derived from a variety of mammalian species were utilized: human (A549 and MRC5) monkey (Vero), mouse (BalB/C), rabbit (RK13), dog (MDCK) and pig (PKIBRS2). These cells were obtained from the European Collection of Cell Cultures (ECACC) with the exception of the PKIBRS2 cells, which were obtained from the Institute of Animal Health (UK) [15]. Derivatives of the A549 cells were used; A549/pr(IFN-β).GFP and A549/pr(ISRE).GFP reporter cell-lines which contain a eGFP (enhanced Green Fluorescent Protein) gene under the control of the IFN-β promoter or an IFN stimulated response element (ISRE) respectively [16], A549/BVDV-NPro and A549/PIV5-V cell-lines which constitutively express the IFN antagonists BVDV-Npro and PIV5-V respectively [14], [17]. A derivative of the MRC5 cell-line that constitutively expresses PIV5-V (MRC5/PIV5-V) was also used [11]. All cell-lines were grown in Dulbecco's modified Eagle's medium (DMEM) supplemented with 10% v/v fetal bovine serum. Viruses used in the study were Bunyamwera wildtype (BUN-WT) and a ΔNSs derivative (BUNΔNSs) [18], Respiratory Syncytial Virus (RSV) and ΔNS1 or ΔNS2 derivatives [7], [19], Influenza (A/PR/8/34) and ΔNS1 derivative [20], Measles (MeV) Edmonson and Mumps (MuV) Enders vaccine strains (NIBSC), and a VΔC derivative of PIV5 (PIV5VΔC) [21]. All viruses were grown and titrated under appropriate conditions.

### IFN induction and signaling reporter assays

The A549/pr(IFN-β).GFP and A549/pr(ISRE).GFP reporter cell-lines were used to test the effect of IFN inhibitors on IFN induction or IFN signaling. A549/pr(IFN-β).GFP were seeded into a 96 well plate and media supplemented with a TBK1 inhibitor (BX795, MRT6884 or MRT67307) or the IKK2 inhibitor (TPCA-1) at the indicated concentrations or equivalent volumes of DMSO. Two hours post-inhibitor treatment cells were infected with a stock of PIV5VΔC rich in defective interfering particles (DIs) to optimally activate the IFN induction pathway and expression of GFP under the control of the IFN-β promoter [16]. Eighteen hours post-infection GFP expression was measured using a Tecan Infinite plate reader at excitation/emission 488/518 nm. Cells were fixed with 5% (v/v) formaldehyde and stained with crystal violet staining (0.015% w/v) to monitor cellular cytotoxicity. A549/pr(ISRE).GFP were similarly seeded and media supplemented with a JAK1 inhibitor (Cty387, AZD1480, Ruxolitinib or Tofacitinib) at the indicated concentrations or equivalent volumes of DMSO. Two hours post-inhibitor addition cell supernatant was supplemented with 10^4^ units/ml of purified α-IFN (Roferon, NHS) to activate the IFN signaling pathway and GFP expression from the IFN response (ISRE) promoter. Forty-eight hours post-IFN stimulation GFP expression and cellular cytotoxicity were measured as described above. Each assay was conducted in triplicate and the mean and standard deviation (StDev) determined.

### Virus plaque assays and growth kinetics

Standard plaque assays were conducted in the appropriate cells using a 0.6% (w/v) avicell overlay [22] and fixed with 5% (v/v) formaldehyde at the indicated times. Plaques were visualized by crystal violet staining (0.015% w/v) or immunostaining using the following primary antibodies; anti-Bunyamwera N protein (Kind gift of Richard Elliott, University of Glasgow), anti-RSV F protein (Serotech), anti-Influenza X31 antibody (Diagnostic Scotland), anti-MeV NP (Abcam) and anti-MuV NP (Abcam) followed by the appropriate alkaline phosphatase conjugated secondary antibody and SIGMA FAST BCIP/NBT substrate. Virus growth kinetics was preformed in A549, A549/BVDV-Npro, A549/PIV5-V, MRC5, MRC5/PIV5-V and Vero cells infected with BUNΔNSs at a 0.001 MOI and grown in the presence or absence of inhibitor at the indicated concentrations. At various times post infection the amount of infectious virus in the culture medium was estimated (pfu/ml) by plaque assays on Vero cells. All experiments were performed at least in duplicate.

## Results and Discussion

**Figure 1:**
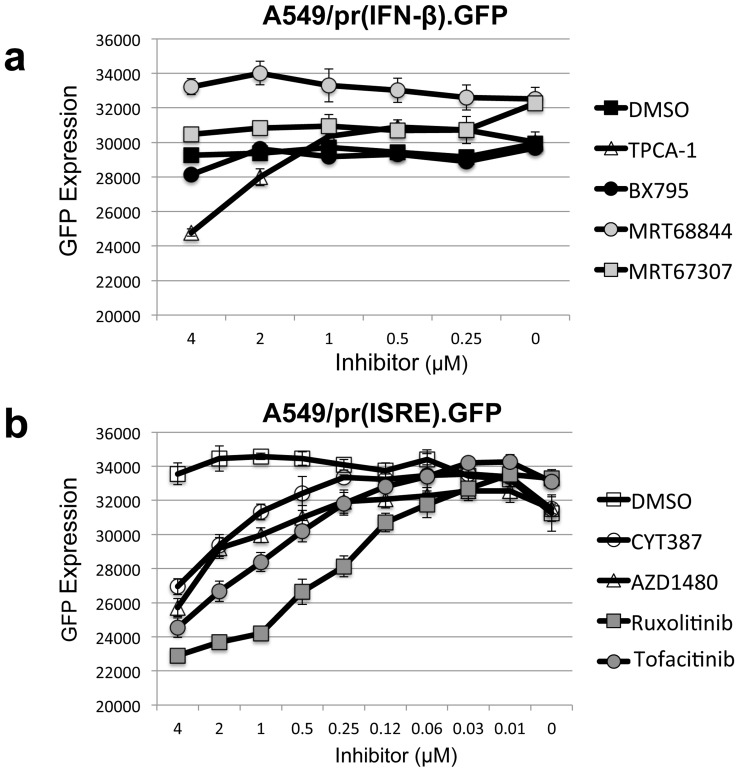
Verification of IFN inhibitors ability to block IFN induction or IFN signaling. (a) Small molecules reported to inhibit the IKK2 (TPCA-1) and TBK1 (BX795, MRT6884, MRT6707) components of the IFN induction pathway were tested using the A549/pr(IFN-β).GFP reporter cell-line which contains an eGFP gene under control of the IFN-β promoter. The IFN induction pathway and hence GFP expression was activated by infection with a DI rich stock of PIV5VΔC. Effect of inhibitors at various concentrations was measured by monitoring fluorescence at 18 hours post-infection. (b) Small molecules reported to inhibit the JAK1 (Cyt387, AZD1480, Ruxolitinib, Tofacitinib) component of the IFN signaling pathway were tested using the A549/pr(ISRE).GFP reporter cell-line which contains an eGFP gene under control of an ISRE promoter. The IFN signaling pathway and hence GFP expression was activated by supplementing the cell supernatant with purified IFN. Effect of inhibitors at various concentrations was measured by monitoring fluorescence at 48 hours post IFN stimulation.

Eight small molecules that have previously been described to inhibit the cellular IFN response were obtained; four inhibitors that target components of the IFN induction pathway: TBK1 inhibitors BX795, MRT68844, MRT67307 [23], [24] and the IKK-2 inhibitor TPCA-1 [25], plus four inhibitors that target JAK1 a component of the IFN signaling pathway: Cyt387, AZD1480, Ruxolitinib and Tofacitinib [26]–[29]. We verified the ability of these molecules to inhibit IFN induction or IFN signaling using two A549 reporter cell-lines in which a GFP gene is placed under the control of either the IFN-β promoter (A549/pr(IFN-β).GFP) or an ISRE promoter (A549/pr(ISRE).GFP) [16]. The four inhibitors targeting components of the IFN induction pathway (BX795, MRT68844, MRT67307 and TPCA-1) were tested using the A549/pr(IFN-β).GFP reporter cell-line. The IFN induction pathway and hence GFP expression was optimally activated in this cell-line by infection with a PIV-5/VΔC virus stock rich in DIs (Fig. 1A). Inhibitors that target components of the IFN induction pathway would be expected to block GFP expression. The IKK-2 inhibitor TPCA-1 demonstrated a significant block to GFP expression, while the TBK1 inhibitor BX795 showed a weak effect at a concentration of 4 µM, however no activity was observed for the MRT68844 and MRT67307 inhibitors (Fig. 1A). The JAK1 inhibitors (Cyt387, AZD1480, Ruxolitinib and Tofacitinib) were similarly tested in the A549/pr(ISRE).GFP reporter cell-line following activation of the IFN signaling pathway using purified IFN (Fig. 1B). All four JAK1 inhibitors blocked GFP expression in the (A549/pr(ISRE).GFP reporter cell-line, however Ruxolitinib had the greatest effect (Fig. 1B). Therefore six of the molecules tested (TPCA-1, BX795, Cyt387, AZD1480, Ruxolitinib and Tofacitinib) inhibited the IFN induction or IFN signaling pathway as expected without causing cellular cytotoxicity (data not shown), however the two MRT molecules (MRT68844 and MRT67307) did not show any activity in this cell-based assay.

**Figure 2:**
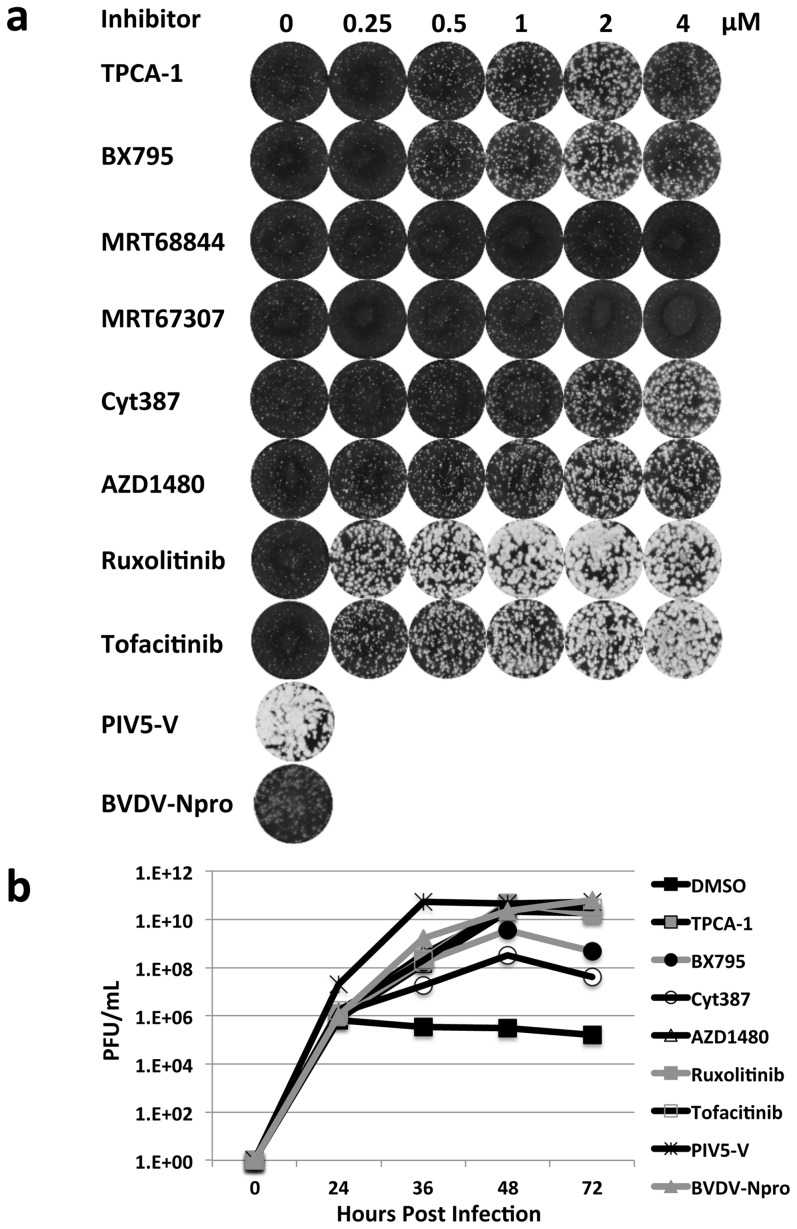
Effect of a panel of IFN inhibitors on BUNΔNSs virus growth in A549 cells. The inhibitor panel consists of small molecules that target the IKK2 (TPCA-1) and TBK1 (BX795, MRT6884, MRT6707) components of the IFN induction pathway and JAK1 (Cyt387, AZD1480, Ruxolitinib, Tofacitinib) a component of the IFN signaling pathway. Effect of the inhibitors was compared with A549 cells constitutively expressing viral IFN antagonists that block IFN production (BVDV-Npro) or IFN signaling (PIV5-V). (a) Effect of various inhibitor concentrations on plaque size formation. Plaques were visualized 2 days post-infection by crystal violet staining. (b) Virus growth monitored over time in presence of 2 µM inhibitor or the equivalent volume of DMSO and pfu/ml determined from reserved supernatants by titration in Vero cells.

Effect of the inhibitors on viral plaque formation was examined using A549 cells infected with recombinant Bunyamwera virus (BUNΔNSs), in which the NSs IFN antagonist has been inactivated rendering the virus IFN sensitive [18]. BUNΔNSs represents a convenient test virus and pathogenic members of the *Bunyaviridae* family are being developed as attenuated vaccines via NSs knockout [5], [6]. Standard plaque assays were performed and fixed 2 days post-infection. A dose-dependent increase in plaque size was observed for all inhibitors with the exception of MRT68844 and MRT67307, which had no effect (Fig. 2). The lack of phenotypic effect observed for the MRT68844 and MRT67307 inhibitors corresponds to their inability to inhibit the IFN induction cascade in our cell-based reporter assay (Fig. 1A). The JAK1/2 inhibitor Ruxolitinib had the most substantial effect; at ≥1 µM plaque formation was equivalent to that observed in A549/PIV5-V cells. The A549/PIV5-V cell-line constitutively expresses the PIV5 IFN antagonist V, which blocks IFN signaling by targeting STAT1 for proteasome-mediated degradation. Growth of IFN-sensitive viruses is boosted in this cell-line [11] and hence it is used here as a control for assessing the effect of inhibitor treatment.

The six effective inhibitors were used to examine their effect on BUNΔNSs growth kinetics and all six inhibitors significantly improved virus titers (Fig. 2b). At 48 hours post-infection titers of BUNΔNSs were ∼5logs greater in the presence of Ruxolitinib, Tofacitinib, AZD1480 and TPCA-1 compared to DMSO treatment. The maximum titer achieved was equivalent to that reached in A549/PIV5-V cells, although for reasons that currently are unclear in the A549/PIV5-V cells the maximum titer was achieved slightly earlier at 36 hours post-infection. It is also noteworthy that the TBK1 and IKK2 inhibitors exhibited a more pronounced effect on boosting viral growth than inhibition of the IFN induction pathway (Fig. 1 and 2). One explanation for this discrepancy maybe that these inhibitors target other cellular components that are not accounted for in the A549/pr(IFN-β).GFP reporter cell-line assay but which have a synergistic effect on boosting virus growth. In conclusion, supplementing cell culture medium with a variety of IFN inhibitors that target different components of the IFN response (TBK1, IKK2, JAK1) significantly boosts replication and yield of an IFN-sensitive Bunyamwera virus.

**Figure 3:**
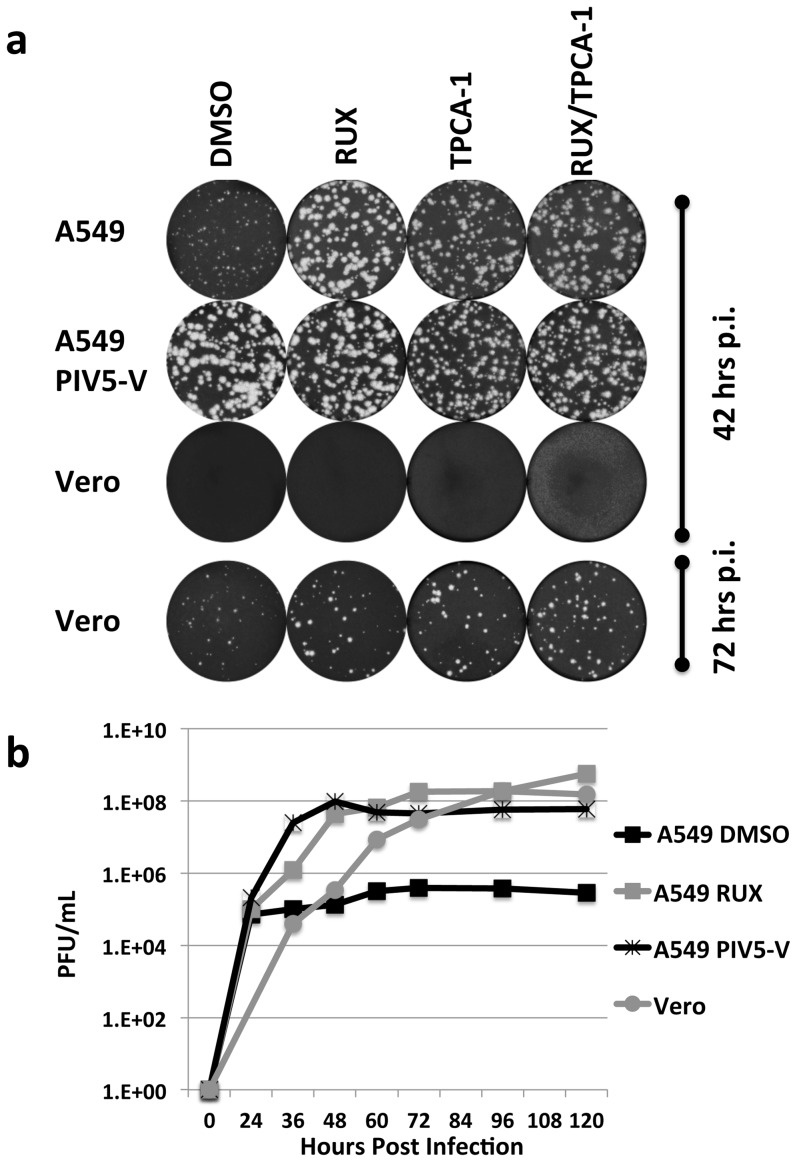
Effect of a combination of different IFN inhibitors on BUNΔNSs growth in A549 and Vero cells. (a) BUNΔNSs plaque formation in A549 or Vero cells in the presence of TPCA-1 or Ruxolitinib (RUX), TPCA-1 and RUX in combination with each other or PIV5-V. Each inhibitor was used at 2 µM and compared to the equivalent volume of DMSO when the inhibitors were used in combination. Plaque size formation was assessed 42 hours post infection (p.i) in A549 cells and both 42 and 72 hours p.i. in Vero cells. Plaques were visualized by crystal violet staining. (b) BUNΔNSs virus growth monitored over time in A549 cells in the presence of 4 µM RUX, the equivalent volume of DMSO or PIV5-V and Vero cells. Supernatants were reserved and pfu/ml determined by titration in Vero cells.

We next sought to determine if two inhibitors targeting different components of the IFN response could further boost BUNΔNSs growth if used in combination with each other or to supplement the medium of infected A549/PIV5-V cells. The IKK-2 inhibitor TPCA-1 and JAK1/2 inhibitor Ruxolitinib were tested. However combinations resulted in no further increases in plaque size in A549 cells and in fact a small decrease in plaque size was observed when the inhibitors were used in combination compared to Ruxolitinib or PIV5-V expressing cells alone (Fig. 3a). One possible explanation for the small decrease in plaque size might be that low levels of cellular cytotoxicity occur in the presence of a combination of inhibitors. The lack of an increase in plaque size suggests that a combination of inhibitors would not lead to significant differences in virus growth kinetics or yield, therefore experiments to test this were not performed. In Vero cells, the two inhibitors, singularly or in combination, did not effect BUNΔNSs plaque size formation (Fig. 3a). However it was of note that BUNΔNSs plaque development was significantly slower in Vero cells compared to A549/PIV5-V cells or to A549 cells supplemented with each inhibitor (Fig. 3a). Growth curves also demonstrated that BUNΔNSs replicated more quickly in A549 cells than Vero cells; at 48 hours post-infection virus titer was significantly higher (∼2log) in A549 cells cultured in the presence of inhibitor or in A549/PIV5-V cells compared to Vero cells (Fig. 3b). The approximate equivalent titer in Vero cells was achieved ∼24 hours later (Fig. 3b). These data support our previous observations suggesting that the default Vero cell-line may not always be the best option to produce the maximum virus yield in the minimum time, presumably due to host cell constraints other than the IFN response that contribute to restricted virus replication [11]. Instead we show that simply supplementing tissue culture media with an IFN inhibitor provides a simple, effective and flexible alternative to facilitate the growth of IFN-sensitive viruses in a cell-line of choice.

**Figure 4:**
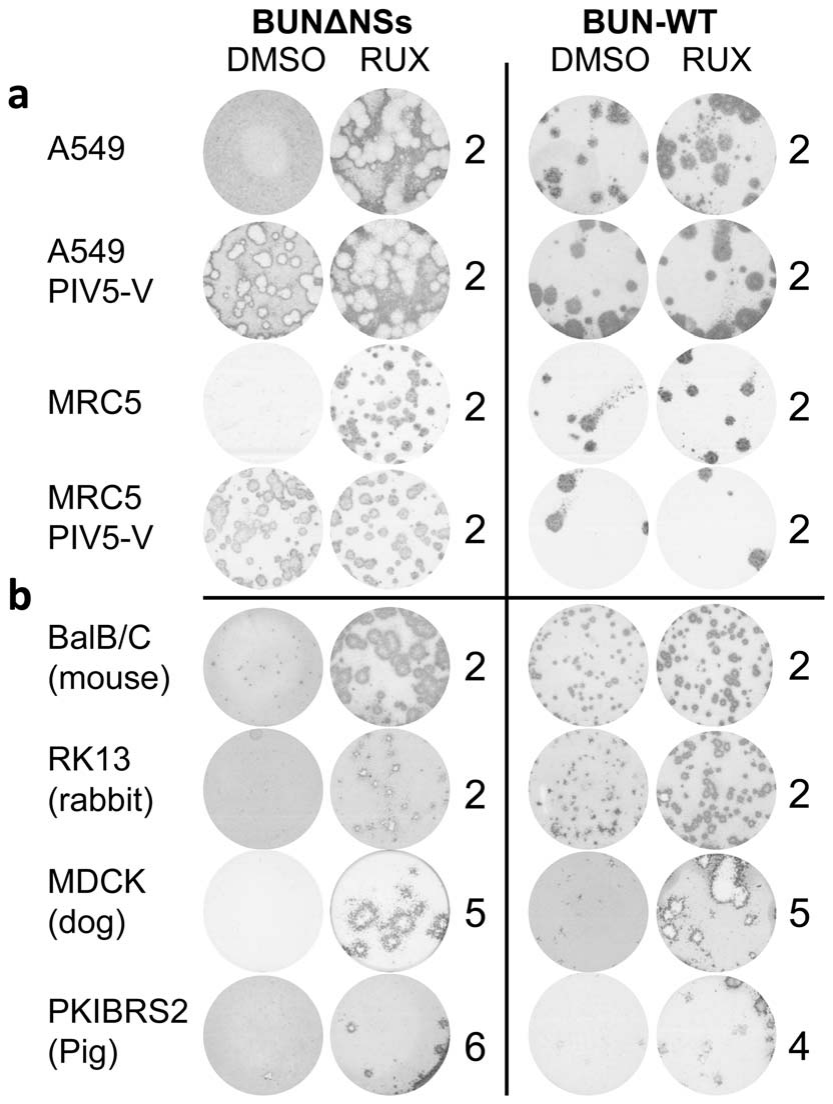
Effect of Ruxolitinib (RUX) on BUNΔNSs and BUN-WT (wildtype) plaque formation in cell-lines derived from different mammalian species. (a) Human cell-lines A549 and MRC5 and their derivatives constitutively expressing the viral IFN antagonist PIV5-V and (b) cell-lines derived from mouse (BalB/C), Rabbit (RK13), Dog (MDCK) and Pig (PKIBRS2). RUX was used at 4 µM and compared with an equivalent volume of DMSO. Plaques were fixed on the day indicated and visualized by immunostaining with an anti-Bunyamwera N protein antibody.

A limited number of cell-lines have regulatory approval for vaccine manufacture e.g. MRC5 [30]. Therefore we extended our study to demonstrate that in MRC5 cells, BUNΔNSs plaque size was increased in the presence of Ruxolitinib and plaques formed were equivalent in size to those in MRC5/PIV5-V cells (Fig. 4a). We further extended our study to examine the effect of Ruxolitinib on plaque size formation in cell-lines derived from different mammalian species using BUNΔNSs and wild-type Bunyamwera virus (BUN-WT) as test viruses. As expected, Ruxolitinib increased BUNΔNSs plaque size to varying degrees in all cell-lines tested (Fig. 4). Ruxolitinib did not significantly affect BUN-WT plaque size in either MRC5 or A549 cells (Fig. 4a). This was not surprising since BUN-WT virus encodes a functional IFN antagonist and can infect humans. However, in mouse- and rabbit-derived cell-lines, BUN-WT formed only small plaques 2 days post-infection and Ruxolitinib moderately increased plaque size (Fig. 4b). More strikingly, BUN-WT formed tiny plaques in dog- and pig-derived cell-lines (Fig. 4b) but Ruxolitinib significantly increased BUN-WT plaque size in these cells (Fig. 4b). One explanation for this data is that the Bunyamwera virus NSs protein is non-functional in dog- and pig-derived cell-lines, suggesting possible species constraints on IFN antagonist function. These results illustrate that use of IFN inhibitors may offer a general approach to quickly initiate studies to investigate species-specific constraints on viral IFN antagonist function, and hence presumably on virus host range. The data also support the concept that supplementing cell-culture medium with IFN inhibitors provides a flexible method to improve techniques to isolate emerging viruses by aiding virus growth in a range of cell-lines derived from different species.

**Figure 5:**
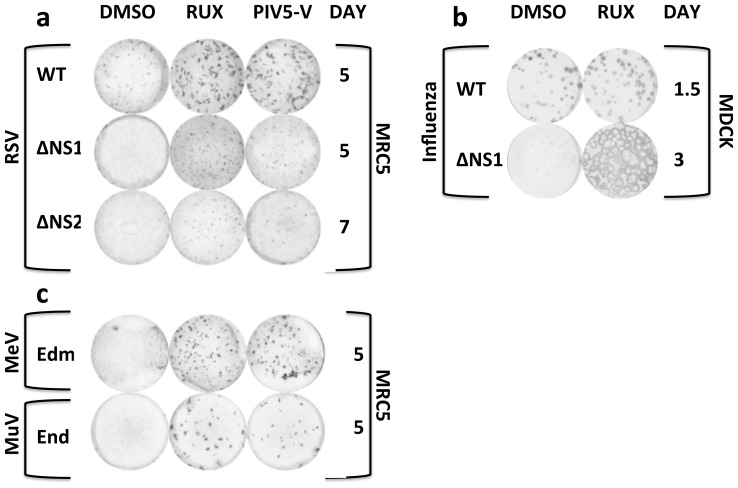
Effect of Ruxolitinib (RUX) on plaque formation of a selection of viruses. (a) RSV WT and derivatives with deleted IFN antagonists NS1 (ΔNS1) and NS2 (ΔNS2). RSV plaques were grown in the MRC5 cell-line or derivative constitutively expressing PIV5-V, fixed on the days indicated and visualized by immunostaining with an anti-RSV F protein antibody. (b) Influenza (A/PR/8/34) WT and derivative with a deleted IFN antagonist NS1 (ΔNS1). Influenza plaques were grown in the MDCK cell-line, fixed on the days indicated and visualized by immunostaining with an anti-influenza X31 antibody. (c) MeV Edmonston (Edm) and MuV Enders (End) live-attenuated vaccine strains. MeV and MuV plaques were grown in MRC5 cell-line or derivative constitutively expressing PIV5-V fixed on the day indicated and visualized by immunostaining with an anti-MeV NP or anti-MuV NP antibodies respectively. RUX was used at 4 µM and compared with the equivalent volume of DMSO.

Respiratory Syncytial Virus (RSV) and Influenza are examples of viruses currently being developed as IFN-sensitive attenuated vaccine candidates [2]–[4], [7], [8]. Deletion of RSV IFN antagonists NS1 and NS2 impairs virus growth in MRC5 cells (Fig. 5a) but Ruxolitinib increased plaque size formation in both viruses to that equivalent of MRC5/PIV5-V cells (Fig. 5a). Therefore IFN inhibitors could be useful in the industrial production of IFN-sensitive attenuated RSV vaccine candidates particularly in light of our previous data demonstrating that higher yields of RSV can be achieved in human-derived PIV5-V expressing cells rather than Vero cells [11]. In addition the ability to grow RSV in a cell-line other than Vero cells could be important for vaccine production because virions produced from Vero cells contain a C-terminally truncated 55KDa G glycoprotein which is responsible for a significant reduction (600-fold) in initial infectivity particularly in primary respiratory epithelial target cells [31]. Therefore the use of IFN inhibitors to facilitate the production of candidate RSV vaccines in a cell-line other than Veros would not only increase virus yield but could also reduce the required vaccine inoculum. Plaque size of wild-type (WT) RSV also increased in the presence of Ruxolitinib (Fig. 5a). This supports our previous observation that inhibiting the IFN response aids the growth of some intrinsically slow growing viruses [11] and could potentially facilitate more rapid isolation of viruses from clinical viral samples. Wild-type influenza (A/PR/8/34) virus plaque size was not increased by Ruxolitinib, presumably because Influenza virus is a fast growing virus that encodes a powerful IFN antagonist the NS1 protein (Fig. 5b). However, Ruxolitinib significantly increased the plaque size of a recombinant A/PR/8/34 ΔNS1 virus that does not encode NS1 (Fig. 5b).

We also tested two traditional vaccine strains, measles (MeV) Edmonson and the Mumps (MuV) Enders, which have been generated empirically using nonsystematic attenuation methods [32], [33]. Plaque size of the MeV and MuV vaccine strains were significantly increased in the presence of Ruxolitinib (Fig. 5c). MeV vaccine strains contain attenuating mutations in the P, V and C proteins that contribute to IFN antagonism [34]. However, MuV Enders contains a functional V protein IFN antagonist [35], providing evidence that IFN inhibitors can boost the yield of viruses with reduced replication rates due to attenuating mutations that do not affect viral IFN antagonists, presumably due to the balance between kinetics of virus replication and induction of the IFN response. This is in agreement with our previous work, which demonstrated that RSV viruses with mutations in G and SH proteins whose functions are not directly relevant to the IFN response grew better in PIV5-V expressing cells [11].

**Figure 6:**
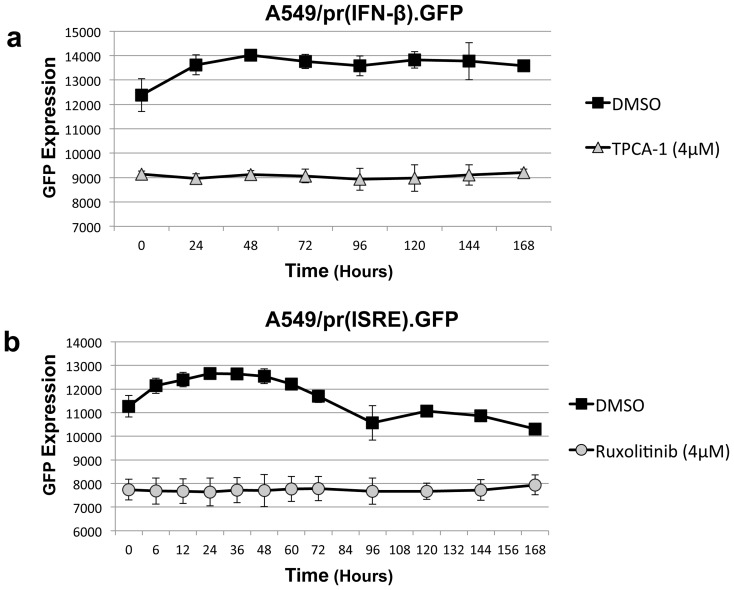
Inhibitory activity of Ruxolitinib and TPCA-1 is stable over time in cell culture. A549 cells cultured at 37°C in the presence of media supplemented with 4 µM Ruxolitinib, 4 µM TPCA-1 or the equivalent volume of DMSO. Cell culture medium was sampled over time and stored at −80°C prior to testing the inhibitory activity of the cell culture medium containing (a) TPCA-1 in the A549/pr(IFN-β).GFP reporter cell-line or (b) Ruxolitinib in the A549/pr(ISRE).GFP reporter cell-line. The reporter assays were conducted using the standard method, briefly the A549/pr(IFN-β).GFP cell-line was activated by PIV5VΔC infection and GFP measured 18 hours post-infection and the A549/pr(ISRE).GFP cell-line activated with purified IFN and GFP measured 48 hours post-IFN treatment.

We have demonstrated that several IFN inhibitors increased virus growth *in vitro*. In the initial plaque formation screen the JAK1/2 inhibitor Ruxolitinib was the most effective and hence was taken forward for further study. Moreover, all the results obtained for Ruxolitinib were essentially mirrored with the IKK2 inhibitor TPCA-1 (data not shown). The plaque assays and growth curves performed required incubation with the inhibitor for multiple days. To ensure our results were not affected by loss of activity of the drug, we used the A549/pr(IFN-β).GFP and A549/pr(ISRE)GFP reporter cell-lines to measure the activity of the drug over time; confirming that the inhibitory effect of both Ruxolitinib and TPCA-1 was stable up to at least 7 days in tissue-culture (Fig. 6).

These results provide proof of principle that supplementing tissue-culture medium with IFN inhibitors provides a simple, effective and flexible approach to enhance virus growth in cell-lines of choice. IFN inhibitors targeting different components of the IFN response (TBK1, IKK2 and JAK1) significantly increased replication and yield of a variety of viruses; including examples of potential vaccine candidates with disabled IFN antagonists (RSV, Influenza), traditionally attenuated vaccine strains (MeV, MuV) and wild-type viruses (RSV, Bunyamwera). In addition JAK1 inhibitors have recently been shown to enhance the growth of oncolytic vesicular stomatitis virus (VSV) in cancer cells resistant to oncolysis [36]. All the viruses tested have an RNA genome, however it is reasonable to predict that IFN inhibitors would also be beneficial in improving the growth of IFN-sensitive viruses harboring a DNA genome. Thus, the use of IFN inhibitors to enhance virus growth *in vitro* can facilitate both basic research and a number of practical applications including vaccine and oncolytic virus manufacture, virus diagnostics and techniques to isolate newly emerging viruses. Potential drawbacks for the use of inhibitors for some or all of the suggested applications are (i) the cost of the inhibitor and (ii) the harvested virus stocks will contain the inhibitor which may affect experiments to address both basic science questions and regulatory problems for medical applications in humans, although in this latter regard it is noteworthy that Ruxolitinib is approved for clinical treatment of myelofibrosis [37]. Purification of virus stocks would eliminate the second issue, and regardless of inhibitor presence, should always be considered for fundamental studies, as a variety of different cytokines that are induced and secreted in response to virus infection will be present in unpurified virus stocks. Since inhibitors such as Ruxolitinib can be administered *in vivo*, they may also prove useful in studies designed to investigate the importance of the IFN response in controlling virus infections in animal models.

## References

[1] Randall RE, Goodbourn S (2008) Interferons and viruses: an interplay between induction, signalling, antiviral responses and virus countermeasures. J Gen Virol 89: 1–47. 18089727 10.1099/vir.0.83391-0

[2] Talon J, Salvatore M, O'Neill RE, Nakaya Y, Zheng H, et al (2000) Influenza A and B viruses expressing altered NS1 proteins: A vaccine approach. Proc Natl Acad Sci U S A 97: 4309–4314. 10725408 10.1073/pnas.070525997 PMC18238

[3] Steel J, Lowen AC, Pena L, Angel M, Solorzano A, et al (2009) Live attenuated influenza viruses containing NS1 truncations as vaccine candidates against H5N1 highly pathogenic avian influenza. J Virol 83: 1742–1753. 19073731 10.1128/JVI.01920-08 PMC2643794

[4] Mossler C, Groiss F, Wolzt M, Wolschek M, Seipelt J, et al (2013) Phase I/II trial of a replication-deficient trivalent influenza virus vaccine lacking NS1. Vaccine 31: 6194–6200. 24183981 10.1016/j.vaccine.2013.10.061

[5] Bird BH, Albarino CG, Hartman AL, Erickson BR, Ksiazek TG, et al (2008) Rift valley fever virus lacking the NSs and NSm genes is highly attenuated, confers protective immunity from virulent virus challenge, and allows for differential identification of infected and vaccinated animals. J Virol 82: 2681–2691. 18199647 10.1128/JVI.02501-07 PMC2258974

[6] Brennan B, Welch SR, McLees A, Elliott RM (2011) Creation of a recombinant Rift Valley fever virus with a two-segmented genome. J Virol 85: 10310–10318. 21795328 10.1128/JVI.05252-11 PMC3196426

[7] Teng MN, Collins PL (1999) Altered growth characteristics of recombinant respiratory syncytial viruses which do not produce NS2 protein. J Virol 73: 466–473. 9847352 10.1128/jvi.73.1.466-473.1999 PMC103853

[8] Luongo C, Winter CC, Collins PL, Buchholz UJ (2013) Respiratory syncytial virus modified by deletions of the NS2 gene and amino acid S1313 of the L polymerase protein is a temperature-sensitive, live-attenuated vaccine candidate that is phenotypically stable at physiological temperature. J Virol 87: 1985–1996. 23236065 10.1128/JVI.02769-12 PMC3571493

[9] Stojdl DF, Lichty BD, tenOever BR, Paterson JM, Power AT, et al (2003) VSV strains with defects in their ability to shutdown innate immunity are potent systemic anti-cancer agents. Cancer Cell 4: 263–275. 14585354 10.1016/s1535-6108(03)00241-1

[10] Naik S, Russell SJ (2009) Engineering oncolytic viruses to exploit tumor specific defects in innate immune signaling pathways. Expert Opin Biol Ther 9: 1163–1176. 19637971 10.1517/14712590903170653

[11] Young DF, Andrejeva L, Livingstone A, Goodbourn S, Lamb RA, et al (2003) Virus replication in engineered human cells that do not respond to interferons. J Virol 77: 2174–2181. 12525652 10.1128/JVI.77.3.2174-2181.2003 PMC140963

[12] Desmyter J, Melnick JL, Rawls WE (1968) Defectiveness of interferon production and of rubella virus interference in a line of African green monkey kidney cells (Vero). J Virol 2: 955–961. 4302013 10.1128/jvi.2.10.955-961.1968 PMC375423

[13] Mosca JD, Pitha PM (1986) Transcriptional and posttranscriptional regulation of exogenous human beta interferon gene in simian cells defective in interferon synthesis. Mol Cell Biol 6: 2279–2283. 3785197 10.1128/mcb.6.6.2279-2283.1986 PMC367773

[14] Hilton L, Moganeradj K, Zhang G, Chen YH, Randall RE, et al (2006) The NPro product of bovine viral diarrhea virus inhibits DNA binding by interferon regulatory factor 3 and targets it for proteasomal degradation. J Virol 80: 11723–11732. 16971436 10.1128/JVI.01145-06 PMC1642611

[15] House C, House JA (1989) Evaluation of techniques to demonstrate foot-and-mouth disease virus in bovine tongue epithelium: comparison of the sensitivity of cattle, mice, primary cell cultures, cryopreserved cell cultures and established cell lines. Vet Microbiol 20: 99–109. 2549683 10.1016/0378-1135(89)90033-3

[16] Chen S, Short JA, Young DF, Killip MJ, Schneider M, et al (2010) Heterocellular induction of interferon by negative-sense RNA viruses. Virology 407: 247–255. 20833406 10.1016/j.virol.2010.08.008 PMC2963793

[17] Killip MJ, Young DF, Gatherer D, Ross CS, Short JA, et al (2013) Deep sequencing analysis of defective genomes of parainfluenza virus 5 and their role in interferon induction. J Virol 87: 4798–4807. 23449801 10.1128/JVI.03383-12 PMC3624313

[18] Bridgen A, Weber F, Fazakerley JK, Elliott RM (2001) Bunyamwera bunyavirus nonstructural protein NSs is a nonessential gene product that contributes to viral pathogenesis. Proc Natl Acad Sci U S A 98: 664–669. 11209062 10.1073/pnas.98.2.664 PMC14645

[19] Teng MN, Whitehead SS, Bermingham A, St Claire M, Elkins WR, et al (2000) Recombinant respiratory syncytial virus that does not express the NS1 or M2-2 protein is highly attenuated and immunogenic in chimpanzees. J Virol 74: 9317–9321. 10982380 10.1128/jvi.74.19.9317-9321.2000 PMC102132

[20] Garcia-Sastre A, Egorov A, Matassov D, Brandt S, Levy DE, et al (1998) Influenza A virus lacking the NS1 gene replicates in interferon-deficient systems. Virology 252: 324–330. 9878611 10.1006/viro.1998.9508

[21] He B, Paterson RG, Stock N, Durbin JE, Durbin RK, et al (2002) Recovery of paramyxovirus simian virus 5 with a V protein lacking the conserved cysteine-rich domain: the multifunctional V protein blocks both interferon-beta induction and interferon signaling. Virology 303: 15–32. 12482655 10.1006/viro.2002.1738

[22] Matrosovich M, Matrosovich T, Garten W, Klenk HD (2006) New low-viscosity overlay medium for viral plaque assays. Virol J 3: 63. 16945126 10.1186/1743-422X-3-63 PMC1564390

[23] Clark K, Plater L, Peggie M, Cohen P (2009) Use of the pharmacological inhibitor BX795 to study the regulation and physiological roles of TBK1 and IkappaB kinase epsilon: a distinct upstream kinase mediates Ser-172 phosphorylation and activation. J Biol Chem 284: 14136–14146. 19307177 10.1074/jbc.M109.000414 PMC2682862

[24] Clark K, Peggie M, Plater L, Sorcek RJ, Young ER, et al (2011) Novel cross-talk within the IKK family controls innate immunity. Biochem J 434: 93–104. 21138416 10.1042/BJ20101701

[25] Podolin PL, Callahan JF, Bolognese BJ, Li YH, Carlson K, et al (2005) Attenuation of murine collagen-induced arthritis by a novel, potent, selective small molecule inhibitor of IkappaB Kinase 2, TPCA-1 (2-[(aminocarbonyl)amino]-5-(4-fluorophenyl)-3-thiophenecarboxamide), occurs via reduction of proinflammatory cytokines and antigen-induced T cell Proliferation. J Pharmacol Exp Ther 312: 373–381. 15316093 10.1124/jpet.104.074484

[26] Pardanani A, Lasho T, Smith G, Burns CJ, Fantino E, et al (2009) CYT387, a selective JAK1/JAK2 inhibitor: in vitro assessment of kinase selectivity and preclinical studies using cell lines and primary cells from polycythemia vera patients. Leukemia 23: 1441–1445. 19295546 10.1038/leu.2009.50

[27] Hedvat M, Huszar D, Herrmann A, Gozgit JM, Schroeder A, et al (2009) The JAK2 inhibitor AZD1480 potently blocks Stat3 signaling and oncogenesis in solid tumors. Cancer Cell 16: 487–497. 19962667 10.1016/j.ccr.2009.10.015 PMC2812011

[28] Quintas-Cardama A, Vaddi K, Liu P, Manshouri T, Li J, et al (2010) Preclinical characterization of the selective JAK1/2 inhibitor INCB018424: therapeutic implications for the treatment of myeloproliferative neoplasms. Blood 115: 3109–3117. 20130243 10.1182/blood-2009-04-214957 PMC3953826

[29] Changelian PS, Flanagan ME, Ball DJ, Kent CR, Magnuson KS, et al (2003) Prevention of organ allograft rejection by a specific Janus kinase 3 inhibitor. Science 302: 875–878. 14593182 10.1126/science.1087061

[30] Jacobs JP, Jones CM, Baille JP (1970) Characteristics of a human diploid cell designated MRC-5. Nature 227: 168–170. 4316953 10.1038/227168a0

[31] Kwilas S, Liesman RM, Zhang L, Walsh E, Pickles RJ, et al (2009) Respiratory syncytial virus grown in Vero cells contains a truncated attachment protein that alters its infectivity and dependence on glycosaminoglycans. J Virol 83: 10710–10718. 19656891 10.1128/JVI.00986-09 PMC2753119

[32] Enders JF, Levens JH, Stokes J Jr, Maris EP, Berenberg W (1946) Attenuation of virulence with retention of antigenicity of mumps virus after passage in the embryonated egg. J Immunol 54: 283–291. 21001446

[33] Hilleman MR, Buynak EB, Weibel RE, Stokes J Jr, Whitman JE Jr, et al (1968) Development and evaluation of the Moraten measles virus vaccine. Jama 206: 587–590. 5695578

[34] Bankamp B, Takeda M, Zhang Y, Xu W, Rota PA (2011) Genetic characterization of measles vaccine strains. J Infect Dis 204 Suppl 1: S533–548. 21666210 10.1093/infdis/jir097

[35] Young DF, Galiano MC, Lemon K, Chen YH, Andrejeva J, et al (2009) Mumps virus Enders strain is sensitive to interferon (IFN) despite encoding a functional IFN antagonist. J Gen Virol 90: 2731–2738. 19625458 10.1099/vir.0.013722-0 PMC2885035

[36] Escobar-Zarate D, Liu YP, Suksanpaisan L, Russell SJ, Peng KW (2013) Overcoming cancer cell resistance to VSV oncolysis with JAK1/2 inhibitors. Cancer Gene Ther 20: 582–589. 24030211 10.1038/cgt.2013.55 PMC4817541

[37] Vaddi K, Sarlis NJ, Gupta V (2012) Ruxolitinib, an oral JAK1 and JAK2 inhibitor, in myelofibrosis. Expert Opin Pharmacother 13: 2397–2407. 23051187 10.1517/14656566.2012.732998

